# The evolution of insect metamorphosis: a developmental and endocrine view

**DOI:** 10.1098/rstb.2019.0070

**Published:** 2019-08-26

**Authors:** James W. Truman, Lynn M. Riddiford

**Affiliations:** Department of Biology, Friday Harbor Laboratories, University of Washington, Friday Harbor, WA 98250, USA

**Keywords:** Broad, E93, juvenile hormone, Krüppel-homolog 1, pronymph

## Abstract

Developmental, genetic and endocrine data from diverse taxa provide insight into the evolution of insect metamorphosis. We equate the larva–pupa–adult of the Holometabola to the pronymph–nymph–adult of hemimetabolous insects. The hemimetabolous pronymph is a cryptic embryonic stage with unique endocrinology and behavioural modifications that probably served as preadaptations for the larva. It develops in the absence of juvenile hormone (JH) as embryonic primordia undergo patterning and morphogenesis, the processes that were arrested for the evolution of the larva. Embryonic JH then drives tissue differentiation and nymph formation. Experimental treatment of pronymphs with JH terminates patterning and induces differentiation, mimicking the processes that occurred during the evolution of the larva. Unpatterned portions of primordia persist in the larva, becoming imaginal discs that form pupal and adult structures. Key transcription factors are associated with the holometabolous life stages: *Krüppel-homolog 1* (*Kr-h1*) in the larva, *broad* in the pupa and *E93* in the adult. Kr-h1 mediates JH action and is found whenever JH acts, while the other two genes direct the formation of their corresponding stages. In hemimetabolous forms, the pronymph has low Broad expression, followed by Broad expression through the nymphal moults, then a switch to E93 to form the adult.

This article is part of the theme issue ‘The evolution of complete metamorphosis’.

## 1. Introduction

**Figure 1.**
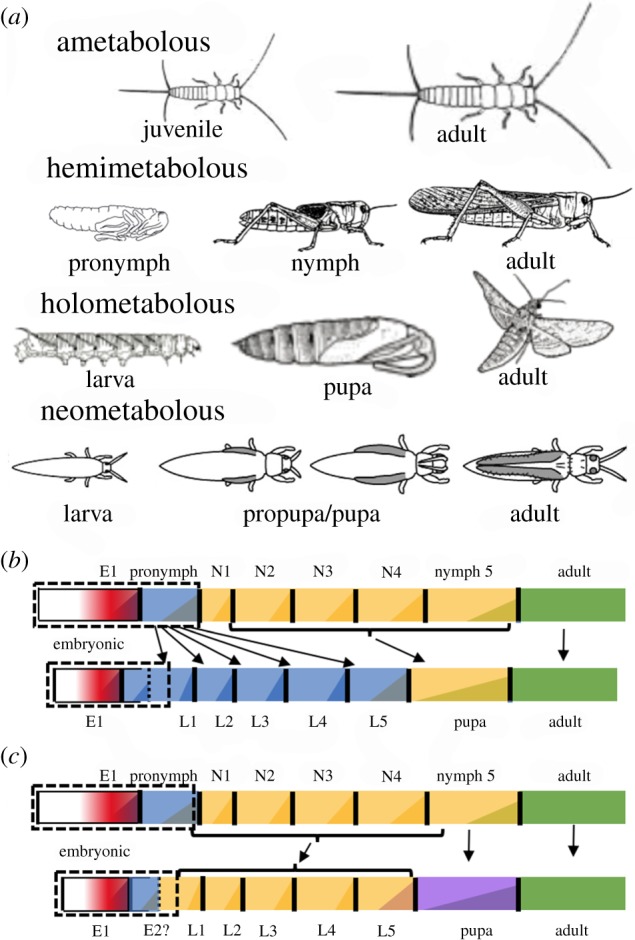
Life-history strategies in the insects. (*a*) Various life-history strategies characterized by silverfish, grasshoppers, sphinx moths and thrips, respectively. (*b* and *c*) The two major hypotheses for the evolution of the holometabolous stages from an unknown hemimetabolous ancestor. In (*b*), the larva arose from conversion of an embryonic stage (the pronymph) into the free-living, feeding larva and the nymphal stages were reduced to a non-feeding, transitional stage, the pupa [2–4]. In (*c*), nymphs and larvae are considered equivalent and the last nymphal instar was modified into the transitional pupal stage [5,6]. (Online version in colour.)

During their evolution, insects have progressed through a number of life-history strategies, some of which persist in present day orders [1] (figure 1). The ancestral strategy was simple direct development, termed *ametabolous* development, as seen in the primitively wingless orders, the Zygentoma (silverfish) and Archaeognatha (jumping bristletails). The juvenile shows very little change as it grows to the adult, and the adult continues to moult in alternation with bouts of reproduction. With the evolution of wings and powered flight, the adult eventually became a terminal stage that no longer moulted, but the immature stage, termed the nymph, usually resembled the adult but lacked wings and genitalia. In its later instars, the nymph bears immobile wing pads that become articulated wings at the moult to the adult. This pattern of development is termed incomplete metamorphosis or *hemimetabolous* development. Hemimetabolous orders include those of the Palaeoptera (Odonata (dragonflies) and Ephemeroptera (mayflies)), the Polyneoptera (the orthopteroid orders including grasshoppers, cockroaches, mantids, termites, stick insects and earwigs) and the Condylognatha (Hemiptera (e.g. true bugs and aphids), Thysanoptera (thrips) and Psocodea (bark lice and true lice)). Although hemimetabolous nymphs generally resemble the adult, the difference between the two stages can be quite dramatic as seen in the transition from the aquatic nymph to the aerial adult in the mayflies and dragonflies. The greatest differences are seen within the Condylognatha, the sister group of the Holometabola [1]. As detailed below, the thrips and some hemipterans have independently evolved a life cycle involving a quiescent stage between the larva and the adult. This condition is referred to as ‘neometaboly’ [7]. *Holometabolous* life cycles include a larval stage that has no resemblance to the adult (indeed, sometimes it is hard to even be recognized as an insect), a non-feeding pupal stage and the adult. This life-history strategy arose in the early Carboniferous period, about 350 Ma, and led to much of the amazing insect diversity that is evident today [8]. The Holometabola includes 11 orders, four of which have been extremely successful: the Coleoptera (beetles), Hymenoptera (ants, wasps and sawflies), Lepidoptera (butterflies and moths) and Diptera (flies and mosquitoes).

The first considerations of the significance of the larva in insect metamorphosis extend back to Aristotle (see [9,10] for a discussion), and there have since been numerous ideas for how the larval and pupal stages of the Holometabola were derived from their hemimetabolous ancestors (summarized in [11]). Two views, though, predominate (figure 1*b*,*c*). The older view is that the larva arose by an arrest of nymphal development during embryogenesis and that the larva essentially represents a free-living, feeding, embryonic stage [2,3]. The larval stage became devoted to growth and the nymphal stages were reduced to a single, non-feeding instar, the pupa, that provided the transition to the adult. The other main view considers that larvae and nymphs are equivalent, and, hence, all immatures are called ‘larvae’. With the progression to the Holometabola, the immature stage had so diverged from the adult plan that the last nymphal instar became converted into the pupal stage to bridge the gap between the two morphologies [5,6,12]. Based largely on observations of developmental endocrinology, we proposed returning to the traditional view distinguishing larvae from nymphs and that the larval form arose by arresting programmes of embryonic development [4,13]. We emphasized that hemimetabolous insects have a cryptic embryonic stage, which we called the pronymph, and that this stage was positioned to evolve into the larva of the Holometabola. Subsequent findings have allowed us to refine this view and are reviewed below.

While there are notable exceptions, such as dragonflies, the typical story within the Hemimetabola is for nymphs and adults to share similar morphologies, habitats and trophic needs. The stunning success of the Holometabola comes from its highly divergent larval stage because it split the life history into two major modules, the larva and adult, that could evolve and adapt independently to exploit different niches for growth versus reproduction [14]. Although there is a great diversity among hemimetabolous nymphs, we think that the shift from the nymph to the holometabolous larva is a qualitative one, rather than just a matter of degree. It is a shift that has not been reversed during the subsequent diversification and expansion within the Holometabola. We will use the terms nymph and larva throughout to denote the immature forms of hemimetabolous versus holometabolous species, respectively, except in the few cases in which a holometabolous-like pattern has evolved within a hemimetabolous group.

## 2. Evolution of the larval form

### (a) Developmental modifications

**Figure 2.**
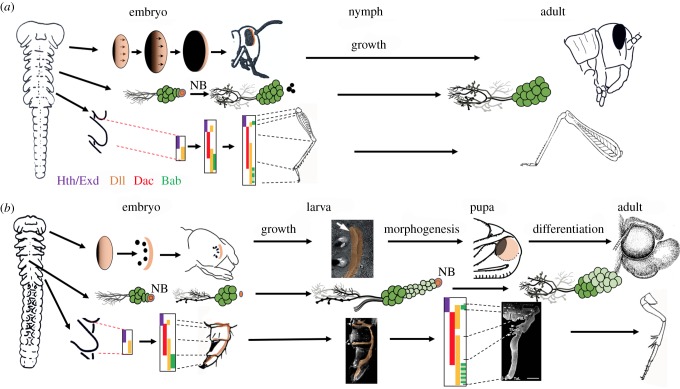
Comparison of embryonic and postembryonic development of a generalized hemimetabolous insect (cricket/grasshopper) with a holometabolous insect (moth). (*a*) Orthopteran development showing progressive patterning of the eye primordium and leg bud. Rows of ommatidia in the eye form as a wave of differentiation (arrows) moves anteriorly across embryonic primordium. CNS neuroblasts (NB) die late in embryogenesis after producing all of their neurons. The leg bud transforms into the leg by the recruitment of a sequence of proximal–distal patterning genes that determine the leg segments. These structures increase in size during nymphal life with little new additions except for ommatidia at the anterior margin of the eye. Based on [15–20]. (*b*) Development in the moth embryo does not progress as far as that in the Orthoptera. Partially patterned systems serve as the basis of larval structures, but persisting embryonic centres (light orange) are carried into the larva and become the imaginal primordia that generate the adult structures. Based on [16–18,21–24]. Hth, Homothorax; Exd, Extradenticle; Dll, Distal-less; Dac, Dachshund; Bab, Bric-a-brac. See text for more details.

Figure 2 compares examples of the embryogenesis of larval and nymphal structures. The central nervous system (CNS) provides an explicit case of the larval organ being an arrested version of the nymphal one. The CNS arises according to a highly conserved developmental ground-plan based on a stereotyped set of stem cells, neuroblasts, each of which generates a characteristic set of neurons [25]. In ametabolous [26] and hemimetabolous groups [15], the neuroblasts generate all of their neurons during embryogenesis; but in embryos of holometabolous orders, they produce only a small number of neurons for the larva and then become dormant. The neuroblasts persist as embryonic stem cells that then reactivate in the larva to make neurons for the adult CNS [16]. Larval and nymphal nervous systems also differ in the features of the neurons that are made during embryogenesis. In grasshoppers, the neurons of the newly hatched nymph already have the basic form and connectivity of the adult cell. In larvae, by contrast, the neurons have modified anatomy and connectivity adapted to larval needs; they only assume their adult form and connectivity when they are remodelled at metamorphosis. The nervous system, then, illustrates two general principles that underlie the generation of the larval form: (i) the arrest of the embryonic developmental programmes and (ii) the redirection of the development of the cells/structures with adaptations that are appropriate for the larval form.

These two principles are also evident in the evolution of the larval eye (figure 2). Larvae typically have a characteristic set of 6–8 lateral single-lensed eyes termed stemmata [17] rather than the complex compound eyes found in nymphs and adults. The eye of the nymph arises from primordia situated on the lateral margins of the embryonic head. The photoreceptor units (ommatidia) first form along its posterior border with successive rows added anteriorly across the primordium. Larvae also begin eye formation at the posterior border of the eye primordium [18], but there are no subsequent additions after the initial photoreceptors are established. The result is either a small compound eye with a few ommatidia (as in scorpionflies) or, more typically, the few photoreceptors are separated and displaced to form the modified stemmata. The anterior portion of the primordium, though, is carried forward to later form the compound eye of the adult [18] (figure 2*b*).

The larval leg is more complex but the message is similar (figure 2). As illustrated by the crickets, *Gryllus bimaculatus* and *Acheta domesticus*, the structure of the leg is established by the sequential recruitment of proximal–distal patterning genes [19,20], starting with *extradenticle* and *Distal-less* promoting the formation of the leg bud and establishing its proximal–distal axis. The following expression of *dachshund* establishes the middle regions of the leg and then *bric-a-brac* appears in the tarsal region in a single domain that later breaks up into subdomains reflecting the tarsal subunits (figure 2*a*). For the legless larvae of *Drosophila melanogaster*, by contrast, the sequence involves only *extradenticle* and *Distal-less.* This arrested system produces Keilen's organ (a sensory remnant of the leg) and embryonic cells set aside for making a future leg [27]. The remainder of the patterning cascade is deferred until metamorphosis when a leg is finally formed. The tobacco hornworm, *Manduca sexta*, provides an intermediate case in which both larva and adult possess functional, but quite different legs [21]. Embryonic patterning of the leg bud progresses through a basal ring of *dachshund* expression and a single *bric-a-brac* domain but then arrests and directs the development of a caterpillar leg [22] (figure 2*b*). As in *Drosophila*, though, the patterning sequence is eventually completed at metamorphosis to make the adult leg.

### (b) The larva and the pronymph

Embryos of hemimetabolous species produce three successive cuticles by the time of hatching [28]. The second embryonic (E2) cuticle belongs to the pronymph and the formation of this stage finishes when the embryo has completely enclosed the yolk. At the pronymph stage, the embryo has undergone sufficient growth and patterning to form a rough approximation of the nymph. There then follows the differentiative growth and maturation that produces the nymph. The patterning programmes that are arrested in making the larva are ones that normally occur during the formation of the pronymph. In addition, except for hatching ‘teeth’, the pronymphal cuticle lacks hardened, sclerotized regions which make it more like typical larval cuticle rather than nymphal cuticle. These features were the basis of our proposal that the pronymph was the likely forerunner of the larval stage [4,13]. In our original hypothesis, we also cited the number of embryonic moults as providing numerical evidence for this correspondence. A subsequent electron microscopic study [28] examined embryos of a number of key species and showed that this direct numerical correspondence was too simplistic. The pronymphal cuticle (which Konopova and Zrzavý [28] call ‘prolarval’ cuticle) was found in all of the embryos of hemimetabolous groups and consisted of epicuticle and lamellar procuticle. However, many holometabolous species also produce three embryonic cuticles [28]. In these cases, though, the E2 cuticle is typically just epicuticle or has loosely packed fibres that may represent the remnants of procuticle. It is either shed at hatching (e.g. Neuroptera and some beetles) or disappears late in embryogenesis (e.g. Lepidoptera). We speculate that the conversion of the pronymph into the larva reduced the need for making three embryonic cuticles and the intermediate cuticle is in the process of being lost. Indeed, in higher Diptera, like *Drosophila*, only two cuticles are formed within the egg.

## 3. The evolution of the pupal stage and of imaginal discs

### (a) Origins of the imaginal primordia

The simplest transition from larva to pupa is illustrated by the abdomen of Lepidoptera. Their larval epidermal cells display different fates at the start of metamorphosis. Highly specialized cells, such as those forming larval hairs and sockets, typically die although a few may survive to make pupal counterparts. Less specialized regions of the epidermis may undergo reduction divisions [29] and there may be localized regions of cell division to make pupal specializations, such as gin traps. Overall, the segment is a fine-grained mosaic of remodelling, proliferation and degeneration as cells are reprogrammed for making the pupa.

Other body regions, though, may show a major replacement of larval cells by adult cells. Indeed, larval organs typically possess cells with latent embryonic potential that is later manifest in making part or all of the adult structure. We call these persisting embryonic regions *imaginal primordia.* As described above for the eye and the leg, these arise from a unitary embryonic primordium, part of which is used to make a larval structure and part of which is carried forward in the larva as an imaginal primordium (figure 2*b*). Such cells are best studied in the epidermis of *Drosophila* in which they comprise small clusters of 20–30 cells termed ‘polyclones’ [27]. Importantly, these persisting embryonic cells may have no larval functions, or they may be an integral part of the larval structure but still retain an embryonic potential that is realized late in larval life.

The spatial relationship of an imaginal primordium to the larval structure may be simple as seen for the eye (figure 2*b*) [18] and the antenna [30], or it can be quite complex as seen for the leg of the caterpillar (figure 2*b*) [21,23]. In the latter, the imaginal leg primordium extends through the larval leg with cell concentrations associated with the major leg regions. These cells make leg cuticle during each larval moult but rapidly grow to make most of the pupal leg at metamorphosis. Interestingly, the beetle, *Tenebrio molitor*, presents a situation in which there appears not to be an organized imaginal primordium [31]. Rather, like the abdomen, its leg appears to undergo a fine-grained cellular transformation from larva to adult.

### (b) The timing of primordia growth and the formation of imaginal discs

**Figure 3.**
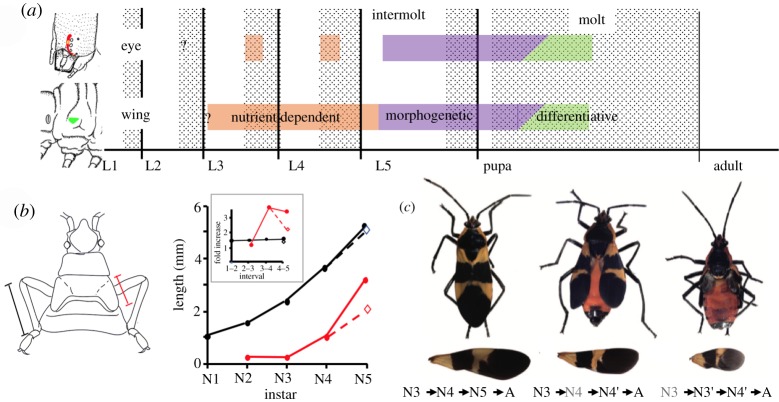
(*a*) Comparison of proliferation in early- and late-forming imaginal discs of *M. sexta.* The eye and wing imaginal primordia go through phases of nutrient-dependent proliferation (orange), morphogenetic proliferation (purple) and differentiative proliferation (green). Both primordia show nutrient-dependent growth in preterminal instars, but proliferation in the eye primordium is restricted to early in larval moults. Both primordia initiate morphogenetic growth early in the last instar and shift to differentiative divisions during the formation of the adult [24,33]. (*b* and *c*) Effects of *br-*dsRNA treatment on patterns of nymphal growth of *O. fasciatus.* (*b*) Growth of the leg (black) and wing pad (red) through the nymphal instars. The leg grows by a constant ratio throughout, but the wing pad shows enhanced growth during the last two nymphal moults. Suppression of Broad expression by injection of *br*-dsRNA in the 4th nymphal (N4) instar redirects the growth of the wing pad in line with the rest of the nymph (open symbol) (data from [34]). (*c*) Pictures of adults and their isolated forewings contrasting an animal going through a normal nymphal series (N#) to adult (A) with nymphs that were injected with *br*-dsRNA at the N4 or N3 instars (green). Subsequent instars repeat the features of the preceding instar and wing growth is suppressed (from [34]). (Online version in colour.)

Based on the extensive work in *Drosophila*, the imaginal primordia that are most familiar are imaginal discs [27]. An imaginal primordium is called an *imaginal disc* when it permanently loses contact with the larval cuticle and ceases to make normal larval cuticle. Imaginal primordia/discs show two phases of growth: in the preterminal larval instars, their growth is *isomorphic* with that of the larval tissues and dependent on nutrient intake. Early in the last larval instar, they switch to the second growth phase, that of *morphogenetic growth*, in which cell divisions are driven by the programmes of tissue patterning and morphogenesis that eventually transform the disc/primordium into its corresponding pupal structure. Once begun, this morphogenetic phase can proceed even if the larva is starved [24,32]. Larvae differ in when their imaginal primordia become imaginal discs [30]. Lepidopteran larvae are especially informative because they have different primordia that use different temporal strategies (figure 3*a*). Primordia for the eyes, antennae and legs are integral parts of the larval structure and secrete new larval cuticle during each larval moult. Their cells divide during the larval moult when they transiently detach from the old larval cuticle. Early in the last larval instar each primordium locally detaches from the overlying cuticle and initiates morphogenetic growth as it transforms into an invaginated disc. In contrast to these *late-forming discs*, the wing primordium is an *early forming disc*. Its cells invaginate in late embryogenesis or early larval life to form an in-pocketed disc that secretes, at best, a very thin cuticle. Freed from making endocuticle, the disc cells can proliferate throughout the intermoult-moult cycle, thereby growing faster than those primordia whose proliferation is locked to the moult. Early forming discs enter into their morphogenetic phase in the last instar along with the late-forming discs (figure 3*a*) [24].

The ancestral condition for the timing of disc formation is probably similar to that seen in the beetles, *T. molitor* [35] and *Tribolium castaneum* [36], in which the wing primordia arise as late-forming discs in the final larval instar. Flies, such as *Drosophila*, are at the other end of the spectrum in that all the adult structures, except the abdomen, come from early forming discs [27]. Early forming discs provide a significant growth advantage, but the ability to form such a disc may be constrained by the complexity of the larval counterpart. For example, in maggots, the larval leg is reduced to a small collection of sensory neurons that provides little impediment to an early forming disc, but for a caterpillar, the space constraints within a complex larval leg might preclude the formation of an early disc.

Regardless of how it forms, a primordium's morphogenetic phase is restricted to the final larval instar in the Holometabola. For nymphs, by contrast, their imaginal primordia are typically just the wing and genital pads, and their morphogenetic growth phase may be spread over a number of nymphal instars. In the milkweed bug, *Oncopeltus fasciatus*, for example, the nymph increases in size by a constant ratio each instar which is reflected in appendages like the legs and antennae increasing in length by 1.5 times for each moult [34] (figure 3*b*). The wing pads show the same progression from N2 to N3, but then enhance their growth during the final two nymphal moults as well as the moult to the adult.

## 4. Developmental hormones and the progression through the life stages

**Figure 4.**
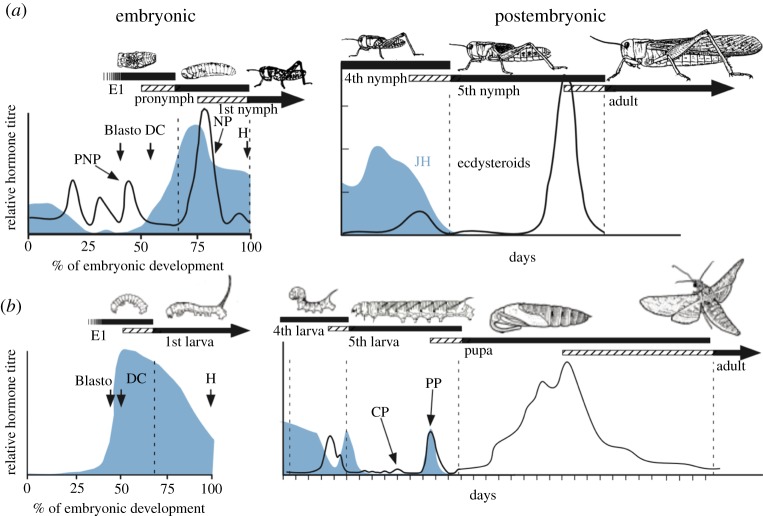
Comparison of the embryonic and postembryonic titres of ecdysteroids (black) and JH (blue) for (*a*) hemimetabolous insects, the grasshoppers *L. migratoria* (embryonic) and *Schistocerca gregaria* (postembryonic) and for (*b*) a holometabolous insect, *M. sexta*. The bars relate the presence of the respective cuticles to the hormone titers (cross-hatching represents pharate periods). Ecdysteroid titres are not available for *Manduca* embryos. Vertical dashed lines: times of ecdysis; Blasto, blastokinesis; DC, dorsal closure; E1, covered by the first embryonic cuticle; H, hatch. Ecdysteroid peaks: CP, commitment peak; PP, prepupal peak; PNP, pronymphal peak; NP, nymphal peak. Reprinted from [4]. (Online version in colour.)

All arthropods rely on surges of steroids to orchestrate the periodic moults that allow for growth and changes in morphology [13] (figure 4). These steroids are ecdysone and its metabolite, 20-hydroxyecdysone (20E), which have differing but overlapping actions. We will refer to these collectively as ecdysteroids. Besides the large moulting peaks of ecdysteroids, smaller peaks can occur during intermoult periods to serve as developmental switches. These low amplitude signals are especially important during the last larval stage of holometabolous insects. While the functions of the ecdysteroids are at least as old as the arthropods, a second family of developmental hormones, the sesquiterpenoid juvenile hormones (JHs) came on the scene more recently (see [37] for a review). In ametabolous species, such as *Thermobia domestica*, JH is primarily involved in the control of reproduction, but it assumes a prominent role in gating the transition from one major stage to the next in insects with more complex life histories.

### (a) Hormonal control of the postembryonic moults

Larvae and nymphs typically go through a characteristic number of instars during their growth and enter metamorphosis when they reach the species-specific threshold size [38]. JH is present through most of this growth but disappears during the last instar as the larva/nymph prepares for metamorphosis (figure 4). In many species, the premature removal of JH induces a corresponding early metamorphosis. Such species are often ones in which the number of instars is plastic and can be adjusted in response to nutritional conditions. Other species, though, such as fly larvae, have a small, invariant number of instars, and this number cannot be changed by either JH removal or continued presence. A reason for such differences is suggested by recent experiments in the silkworm, *Bombyx mori*, using genetic means to prevent JH production or JH action by removing the JH receptor Methoprene-tolerant (Met) or the Krüppel-homolog 1 (Kr-h1) transcription factor that mediates JH action [39,40]. Such larvae progress through the first two larval moults and only attempt precocious metamorphosis after they reach the L3 instar. Similar results are seen in hemimetabolous species after knockdown of the JH receptor or Kr-h1 in the bug *Pyrrhocoris apterus* [39] or the prevention of JH production by treatment of *Locusta migratoria* eggs with allatocidal drugs such as the precocenes [41]. The hatchlings always undergo a couple of nymphal moults before attempting metamorphosis. The data from *Bombyx* suggest that there is a ‘competence factor’ that appears after the first few moults to direct development into a metamorphic pathway [40]. After the competence factor appears, JH is needed for the animal to remain as a larva or a nymph. This arrangement provides plasticity to deal with food deprivation in the later instars by sustaining JH production and being able to intercalate an additional instar to accommodate the required growth. In maggots, by contrast, the larva may already be committed to produce the final larval stage at the time that such a competence factor appears [42]. This would be too late for JH to evoke an additional larval moult.

Pupation involves a switch in cuticle type and also the requisite growth and morphogenesis of imaginal primordia/discs to allow the establishment of the pupal form. In *M. sexta*, the presence of JH in the preterminal larval instars allows the primordia to undergo nutrient-dependent growth but not morphogenetic growth (figure 3*a*) [24]. Although JH is still present in the early part of the last larval instar, a factor associated with feeding on protein switches on the morphogenetic programme in these primordia [24,43]. This factor, called metamorphosis initiation factor (MIF), is probably different from the competence factor mentioned above, but its nature is also unknown, although it can be mimicked by mammalian insulin [44]. Once in their morphogenetic programme, the primordia are committed to the pupal state and can no longer make larval cuticle. The general epidermis, by contrast, retains its larval competence for a few more days until the day before wandering, when a small peak of ecdysteroids, acting in the absence of JH, induces its pupal commitment [45] (figure 4*b*). Proliferation and morphogenesis continue in these tissues for the next few days until the large prepupal peak of ecdysteroids causes the deposition of the pupal cuticle. This prepupal peak is accompanied by the brief return of JH, which acts on imaginal tissues to prevent the precocious production of adult structures. Species differ in the extent to which their tissues require a prepupal JH exposure to prevent premature adult differentiation. In classic studies on the wild silkmoth, *Hyalophora cecropia*, virtually all imaginal structures show premature adult differentiation if JH is not present [46]. In *Manduca*, the overshoot is largely confined to the posterior portion of the eye [33], while, in *Bombyx*, no structures appear to be affected [40]. The final transition from the pupa to the adult then requires ecdysteroids acting in the absence of JH, and the experimental treatment of pupae with JH mimics redirects development back to the pupal state [46,47]. The latter hormonal situation is similar to that seen in hemimetabolous species going from the last stage nymph to the adult (figure 4).

### (b) Hormones and embryonic development

While JH is involved in a complex interplay between tissue morphogenesis and stage-specific cuticle production during the postembryonic period of the Holometabola, its functions during the embryonic period are relatively minor. The most detailed data are from Lepidoptera. Their embryos start making JH after blastokinesis, as the first stage larva is undergoing differentiation and cuticle deposition [40,48] (figure 4*b*). Early treatment of embryos with JH or JH mimics prevents blastokinesis, but the differentiation of the larva is largely normal [13,49]. Conversely, the prevention of JH production by embryos of *B. mori*, by knocking down juvenile hormone acid methyl transferase (JHAMT) (the enzyme that converts JH acid to JH), results in only a slight delay in embryogenesis [40]. Although many larvae do not hatch, they can subsequently feed and grow normally if manually removed from the egg shell.

In contrast to holometabolous species, the embryos of ametabolous and hemimetabolous species are quite sensitive to treatment with exogeneous JH or JH mimics [4,13,20]. In grasshoppers, the three embryonic cuticles are laid down in response to three ecdysteroid pulses within the egg [50] (figure 4*a*). The moults to the E1 stage and to the pronymph (E2) occur in the absence of JH, while the last moult to the nymph occurs in its presence [51]. A similar endocrine pattern is seen in cockroaches [52–54]. In grasshoppers and crickets, exogenous JH has no discernable effects up through the E1 moult, but its appearance during the transition to the pronymph arrests growth and patterning and induces secretion of a nymphal cuticle rather than the pronymphal cuticle [4,20]. The pronymph is converted into an N0 instar whose appendage development varies depending on the time of JH treatment. An attempt to remove JH from the cockroach, *Blattella germanica*, embryos using maternal RNA interference (RNAi) treatment to suppress the production of JHAMT produced variable results but 23% of the treated embryos could not progress beyond the pronymph stage [55]. Whether they simply stopped or underwent a second pronymphal moult rather than the N1 moult was not determined.

These effects of JH treatment on hemimetabolous embryos are strikingly similar to the changes that were needed to convert a nymph into a larva. As discussed above, for the latter to occur, embryonic structures were arrested at intermediate stages of development and differentiated into functional units. Early JH treatment evokes both the embryonic arrest and the premature differentiation of these partially patterned structures. It appears that the embryos only acquire sensitivity to JH treatment after forming their E1 cuticle. Hence, tissue patterning that occurs before this event is JH-insensitive. This relationship might help explain features of the larval structure that are similar across the orders. In the case of the eye, for example, the embryos of the holometabolous ancestor may have started to determine the first photoreceptor units at the posterior region of the eye just before the end of the E1 moult. The premature appearance of JH would still allow them to form (and eventually be modified into stemmata), but the rest of the eye primordium would be suppressed.

## 5. Genetic circuits controlling life-history stages

Three genes, *Kr-h1*, *broad* and *Ecdysone-inducible protein 93F* (*E93*), are key genes that respond to the developmental hormones and control the characteristics of the different life stages. All were initially identified in *Drosophila* and associated with ecdysteroid action during metamorphosis.

*Kr-h1* was first described by Pecasse *et al*. [56] based on the disruption of prepupal development in *Kr-h1* mutants. Although intimately associated with the maintenance of the larval or the nymphal condition, *Kr-h1* is not, strictly speaking, a larval specifying gene. Studies on a number of species show that *Kr-h1* is the main target of JH acting through its receptor Met [57–59], and hence the main effector of JH action. In the flour beetle, *T. castaneum*, for example, the removal of either JH, Met or Kr-h1 produces the same developmental response: the larvae initiate premature metamorphosis [57,60]. Therefore, if JH is required for larval maintenance, then the same is true for Kr-h1. Especially informative in this regard are the *dimolting* (*mod*) mutants in *B. mori*, described above. Despite their lack of JH, they can undergo larval moulting until the L3 instar and they do so despite severely depressed levels of Kr-h1 [39]. When JH reappears late in the larval–pupal transition to prevent the premature adult differentiation of some imaginal primordia, this action is also mediated through Kr-h1 [57,59,61].

Broad is a zinc-finger transcription factor with multiple isoforms, each carrying a different pair of C-terminal C2H2 zinc-fingers, which control their DNA-binding specificity [62]. Isoform numbers range from four in *Drosophila* [62] and Lepidoptera [63,64], five in *Tribolium* [65,66], to six in *Blattella* [67]. *Drosophila* mutants that lack *broad* function go through larval life but are blocked at the entry to metamorphosis [68]. In Diptera and Lepidoptera, *broad* mRNA and protein appear in tissues when they become committed to pupal differentiation. In the general epidermis of *Manduca*, pupal commitment and *broad* expression are induced by 20E acting in the absence of JH on the day before wandering [45,63]; but in the imaginal discs and primordia, *broad* mRNA appears earlier when they shift to morphogenesis [43,63]. Broad then disappears when the pupa begins the transformation to the adult, but the treatment of the pupa with JH allows *broad* to be re-induced by 20E and a second pupal moult ensues [47]. A series of genetic gain-of-function and loss-of-function experiments in *Drosophila* showed that Broad has two functions: (i) it activates genes specific to the pupal stage and (ii) it suppresses both larval- and adult-related programmes [47,62]. This dual action of Broad is strikingly evident in *Tribolium*, in which Broad knockdown by RNAi treatment of the last larval instar results in larval–adult mosaics rather than pupae [65,66]. A similar dual action of Broad is also seen in the neuropteran, *Chrysopa perla* [65].

While *broad* is the stage specifying gene for the pupal stage, *E93* plays this role for the adult [69]. Its role in specifying the adult stage in the Holometabola was first demonstrated in *Tribolium* [69]. It first appears during the prepupal stage and is high during the subsequent formation of the adult. Knockdown of *E93* mRNA results in a repeat of the pupal moult rather than the formation of the adult. The high levels of E93 after pupal ecdysis reduce both *broad* and *Kr-h1* expression, allowing the animal to progress to the adult.

**Figure 5.**
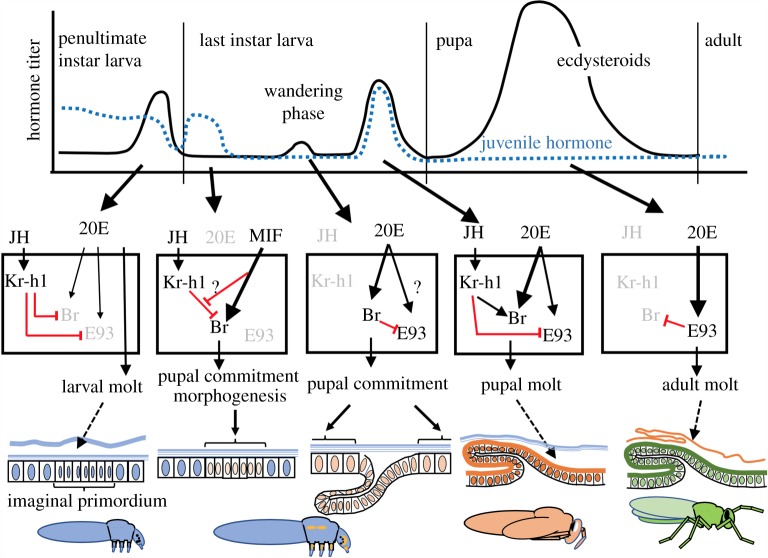
A generalized scheme shows in holometabolous insects how stage specification genes relate to each other, to the hormonal environment that orchestrates metamorphosis and to the cellular responses of the imaginal primordium and the general epidermis. Symbols that are greyed out are either absent or suppressed. See text for details. 20E, 20-hydroxyecdysone; Br, Broad; E93, Ecdysone-inducible protein 93F; JH, juvenile hormone; Kr-h1, Krüppel-homolog 1; MIF, metamorphosis initiation factor. Based on [24,57,59,61,63,65,66,69,70]. (Online version in colour.)

The relationships of *Kr-h1*, *broad* and *E93* relative to formation of the larval, pupal and adult stages of the Holometabola are summarized in figure 5 [57,59,61,63,65,66,69,70]. During the larval instars, JH maintains the larval state through Kr-h1 expression, which also suppresses the expression of *E93* and *broad*. Both Broad and E93 appear during the last larval instar but due to factors that differ for the imaginal primordia versus the general epidermis. In *Manduca*, for example, stage and nutrition-related factors (such as MIF) induce Broad in the imaginal primordia even though JH is still present early in the last instar. Ecdysteroids at wandering then induce Broad in the rest of the epidermis, but only if JH is absent. In *Tribolium*, E93, as well as Broad, may appear as tissues commit to metamorphosis. In this beetle, E93 may provide the metamorphic switch [71] because its loss results in the maintenance of the larval form while the loss of Broad allows metamorphosis to proceed forming a larval–adult mosaic rather than a pupa. Under normal circumstances, JH then returns to suppress *E93* expression and to support *broad* expression, thereby insuring a pupal moult. In the derived development of *Drosophila*, *broad* and *E93* maintain their roles in directing pupal and adult development, respectively, but the control over the entry to metamorphosis has shifted to *broad*, since *broad* null mutants stay as permanent larvae [68] while *E93* null mutants form abnormal pharate adults [72]. For the adult moult, the absence of JH then permits high expression of E93 which, in turn, suppresses *broad* expression and drives adult differentiation [69–73].

Considering their roles in specifying the life stages of Holometabola, the functions of Kr-h1, Broad and E93 in hemimetabolous species has been of great interest [34,39,58,69,74–76]. Two of these genes are relatively consistent in their function. JH effects are mediated through the induction of Kr-h1 in both groups [39,58,74]. Therefore, regardless of whether one considers a larva to larva moult or a nymph to nymph moult, the *status quo* action of JH is mediated through Kr-h1. E93 also has similar roles in both hemimetabolous and holometabolous forms. It appears during the final nymphal stage of hemimetabolous insects to promote adult differentiation [69]. In so doing, it also suppresses Broad, whose expression is a characteristic feature of the nymphal stages. As stressed by Belles & Santos [77], this hemimetabolous pattern is somewhat different from that seen in holometabolous species in which some E93 expression is evident in the prepupa and then becomes very high for the formation of the adult. At least in some imaginal tissues, this early E93 expression is held in check by Broad, because without the latter, the tissue jumps directly to adult differentiation and the animal becomes a larval–adult mosaic.

The central question, then, is what is the role of the pupal specifying gene, *broad*, in hemimetabolous insects that do not have a pupa? *broad* is found in hemimetabolous and ametabolous insects [34,74–76] and even in crustaceans [78,79], but not in other classes of arthropods. Its postembryonic function in hemimetabolous insects was first examined in the milkweed bug, *O. fasciatus* [34]. *broad* transcripts were prominent in nymphal instars, especially during nymph to nymph moults but they then disappeared during the last moult from nymph to adult. Treatment of nymphs with precocene or JH mimics caused premature or delayed metamorphosis, respectively, and had a corresponding effect of advancing or delaying the disappearance of Broad. However, the knockdown of *broad* mRNA in preterminal nymphal instars using RNAi did not cause premature metamorphosis of these bugs [34], nor does it do so in the cockroach *Blattella* [75]. Instead, removal of Broad had two interesting effects in *Oncopeltus*: (i) nymphs normally show instar-specific pigmentation patterns during the last three instars, but without Broad they repeat the character of the preceding instar even though they show normal overall growth, and (ii) the wing pads cannot shift into their morphogenetic mode of growth but rather maintain their growth in concert with nymphal structures like the legs and antennae (figure 3*c*) [34]. *Blattella* does not have obvious instar-specific features that can be analysed, but *broad* knockdown in that species also selectively suppressed wing growth [75], suggesting a similar need for *broad* to support morphogenetic growth of cockroach imaginal primordia. This requirement of Broad to support morphogenetic growth in the imaginal primordia (wing pads) of nymphs has obvious parallels with Broad's appearance in the imaginal primordia and discs of larvae when they shift to their morphogenetic growth phase in preparation for metamorphosis. However, in the Holometabola, this Broad-associated growth phase is confined to the last larval stage in preparation for pupation; whereas in hemimetabolous nymphs, it is spread over a number of the late nymphal instars.

The embryonic expression of *broad* is also informative. There is an expression of some *broad* isoforms during embryogenesis of holometabolous insects, but this expression is largely confined to the CNS and not related to the gene's metamorphic functions [65,80]. In *Blattella*, some Broad isoforms are present in the early embryo but the highest levels of *broad* transcripts occur at embryonic day 8 [67], possibly in response to the small peak of ecdysteroids on day 6 [53], and just as JH is appearing in the embryo [54]. Nymphal differentiation then occurs when both Broad and JH are high [67]. Maternal suppression of either *broad* expression [67] or JH signalling (via knockdown of JHAMT, Kr-h1 or Met [55]) led to a substantial percentage (ranging from 23 to 45%) of embryos being blocked after the formation of the pronymph. The suppression of JH signalling also reduced Broad levels in embryos [55]*.* Elevated levels of JH and Broad are clearly features of nymphal differentiation but more work is needed to resolve the details of this interplay.

## 6. Implications for the evolution of the Holometabola

In this review, we support the century old theory by Berlese [2] that the larval form arose by ‘de-embryonization’, i.e. through the suppression/arrest of ancestral programmes of embryonic development. Similar shifts in the time of hatching relative to an ancestral developmental time-line occur in many groups, the most notable being the shift from precocial to altricial development in birds [81]. For the precocial hatchling, the chick is highly mobile at hatching and capable of foraging (often with parental supervision), but for the newly hatched altricial young many organ systems are only partially developed, resulting in a chick that is completely dependent on its parents for survival. Although only incompletely developed, the altricial hatchling may nevertheless have specialized adaptations, such as brightly coloured patches within their mouth, that enhance their survival. The altricial strategy in insects, though, while involving the suppression of embryonic programmes, does not result in a partially developed, helpless nymph. Rather, the result is a specialized larval form that can live independently from the very moment of hatching. The situation in insects is further complicated by trying to reconcile the gradual developmental processes occurring within the animal to the abrupt, saltatory changes displayed on the animal's surface as it periodically moults its external cuticle and then fitting these changes into discrete life-history categories.

**Figure 6.**
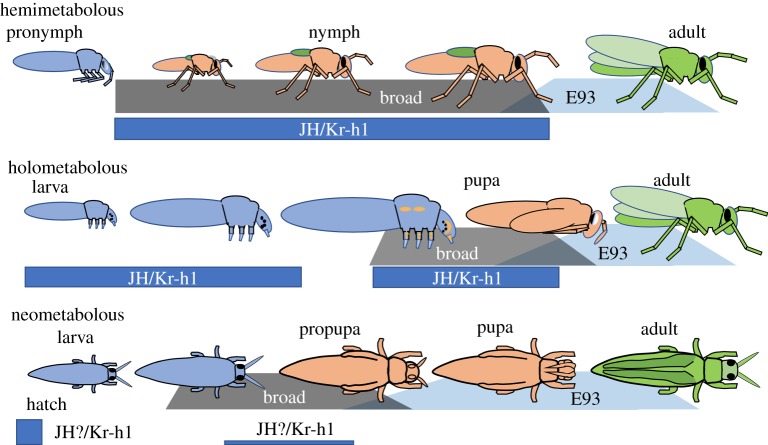
Generalized diagram showing the relationship of Kr-h1, Broad and E93 expression to the various life stages of hemimetabolous, holometabolous and neometabolous insects. Based on [34,63,65,66,69,75,82,83]. See text for details. (Online version in colour.)

Our view of the evolution of the holometabolous life history from an unknown hemimetabolous ancestor is summarized in figure 6 (see also [4,13]). The problem has been one of transforming the two-part life history of nymph and adult into a three-part sequence of larva, pupa and adult. We believe that the hemimetabolous forms also have a three-part life history of pronymph, nymph and adult, although one phase is cryptic within the egg. As described above, each stage has distinctive adaptations reflected in its morphology and cuticle, expression of stage specifier genes, and JH-operated switches for the transformation from one stage to the next. In considering the pronymph as the forerunner of the holometabolous larva, it is significant that the developmental programmes that were arrested to establish the larval form were ones that normally occur during the formation of the pronymph. Also, some pronymphs have adaptations for hatching and/or escape from oviposition sites, and similar adaptations are features of many larvae. For example, the grasshopper pronymph is called the ‘vermiform larva’ [84] and, until the shedding of the pronymphal cuticle, it moves in a ‘larval-like’ way by peristaltic waves along its body rather than by use of its appendages. As we suggested previously [4,13], burrowing adaptations of the pronymph, which allowed it to move through substrates that were unavailable to the nymph or the adult, may have been an important preadaptation for becoming a larva. The pronymph would need an additional change that would allow it to feed in this form, but once achieved, new food resources might become available to it, thereby providing a selective advantage to convert the larva into the main feeding stage and eventually reducing the nymph to a transition stage.

New insights into the various life stages have come from the genes *Kr-h1*, *broad* and *E93* (figure 6). As Kr-h1 mediates the pathway for JH action [39,58,74,77], it appears at any point in the life history when JH is present. This includes nymph to nymph moults and larva to larva moults. JH also transiently appears during the stage change from pronymph to nymph [51,54] and during pupal formation [13], and in both cases, the JH appearance is accompanied by the expression of *Kr-h1*. As described above, the transcription factor *broad* has many isoforms and this gene predates metamorphosis. Its functions are complex and only some of the *broad* isoforms have assumed a role of specifying the pupal stage in the Holometabola, where *broad* is both necessary and sufficient to establish the pupal state. In hemimetabolous insects, by contrast, *broad* expression is a feature of the nymphal stages and detailed studies in the cockroach show a marked upregulation of *broad* isoforms, especially the Z1 isoform that is the principal metamorphic isoform in flies [62], immediately after the formation of the pronymph, as the formation of the nymph is beginning [67]. Therefore, the pronymph and the larva share the feature of low *broad* expression and their transition to their next life stage, the nymph and pupa, respectively, is associated with enhanced *broad* expression, especially of the *br-Z1* isoform. The understanding of how some of the *broad* isoforms evolved the role of specifying the pupal stage requires a better understanding of the role of Broad in the hemimetabolous nymph. *broad* RNA knockdown experiments in *Oncopeltus* [34] and *Blattella* [75] do not support it as having a role as a nymph specifying gene, however, experiments reported in this issue show that *broad* knockdown causes precocious metamorphosis in the cricket *G. bimaculatus* [85]*.* These conflicting results may reflect a difference in the role of *broad* in these species or a difference in the effectiveness of the RNAi knockdown. *broad* knockdown in nymphs has a consistent effect of suppressing the premetamorphic acceleration of growth in the wing pads [34,75] and this region shows elevated expression of *broad*. Possibly, the level of knockdown achieved in some species is sufficient to suppress the growth response of the wing pad but not the stage characteristics of the animal as a whole. These species differences need to be resolved before we can understand how *broad* assumed its role as the pupal specifier gene.

In terms of the association of Broad with the making of the pupa, it is interesting that hemimetabolous insects have made two other attempts to achieve complete metamorphosis that involved evolving a pupal stage. This has occurred independently in the thrips (Thysanoptera) and scale insects and whiteflies (Hemiptera: Coccidae). In the thrips, *Frankliniella occidentalis* and *Haplothrips brevitubus*, Kr-h1 is very high at mid-embryogenesis, similar to that seen in insect embryos in general because of the high levels of JH at the end of embryogenesis. It then falls to moderate to very low levels during the two larval stages with some rebound during the formation of the propupa [82]. *Broad* expression is low in the first larval stage but then rises in the second larval instar in preparation for the production of the propupa (the first pupal instar) and continues for the production of the pupa [82]. Similarly, in males of the Japanese mealy bug *Planococcus kraunhiae*, *broad* expression is evident in the male as it transforms into a prepupa and then a pupa but is absent from the female, which remains in a permanent nymphal condition [83]. Therefore, in these two cases, as in the Holometabola, *broad* expression is a prominent feature of the pupal stage.

The final gene of importance is E93. In hemimetabolous insects, E93 expression causes the juvenile form (the nymph) to transform into the adult [69]. As discussed in a number of papers [61,69,70,77], the relationship of E93 is more complicated in the case of holometabolous species because of the interposition of the pupal stage between the juvenile form (the larva) and the adult. E93 begins to be expressed in the prepupa, but the intervention of Broad expression represses E93 action and directs pupal differentiation. An early propupal expression of E93 is also seen in the neometabolous insects, the thrips (Y. Suzuki, T. Shiotsuki, A. Jouraku, K. Miura, C. Minakuchi 2019, personal communication) and the mealy bug [86].

Without the intervention by Broad in this early E93 action, some larval tissues can jump directly to forming adult structures. The maintenance of Broad and suppression of E93 in these tissues is a result of the prepupal peak of JH. This JH peak is a unique feature of the prepupal period in the Holometabola, but the transient re-expression of Kr-h1 at this time in the Neometabola suggests that they may have a similar JH peak. The evolutionary origin of this prepupal peak is unknown. It is worth remembering, though, that a transient peak of JH is associated with the stimulation of *broad* expression and nymphal differentiation in hemimetabolous embryos. As detailed above (figure 2), the holometabolous larva carries embryonic stem cells and primordia that delay their patterning and differentiation until metamorphosis. The requirement of these tissues for JH in the prepupa may be related to the need that they had for this hormone when these events were confined to the embryonic stage of their ancestors.

It should be cautioned that our conclusions about the overall patterns of endocrinology, development and gene networks within the insects are based on detailed knowledge of only a few species. Insects at key evolutionary nodes, such as dragonflies and mayflies, are virtually unknown from these perspectives. We recognize that our above discussion of how these factors relate to the evolution of metamorphosis is vulnerable because of this shortcoming. Future work will hopefully use information from diverse insect groups to support, refute or modify these ideas to bring about a fuller understanding of how insects acquired the wondrous diversity of life histories that they display.

## Data accessibility

This article has no additional data.

## References

[1] Misof B et al. 2014 Phylogenomics resolves the timing and pattern of insect evolution. Science 346, 763–767. (10.1126/science.1257570) 25378627

[2] Berlese A 1913 Intorno alle metamorfosi degli insetti. Redia 9, 121–136.

[3] Imms AD 1937 Recent advances in entomology. Philadelphia, PA: Blakiston.

[4] Truman JW, Riddiford LM 1999 The origins of insect metamorphosis. Nature 401, 447–452. (10.1038/46737) 10519548

[5] Poyarkoff E 1914 Essai d'une théorie de la nymphe des Insectes Holométaboles. Arch. Zool. Exp. Gen. 54, 221–265.

[6] Hinton HE 1963 The origin and function of the pupal stage. Proc. R. Entomol. Soc. London Ser. A 38, 77–85.

[7] Sehnal F, Švácha P, Zrzavy J 1996 Evolution of insect metamorphosis. In Metamorphosis: postembryonic reprogramming of gene expression in amphibian and insect cells (eds Gilbert LI, Tata JR, Atkinson BG), pp. 3–58. San Diego, CA: Academic Press.

[8] Rainford JL, Hofreiter M, Nicholson DB, Mayhew PJ 2014 Phylogenetic distribution of extant richness suggests metamorphosis is a key innovation driving diversification in insects. PLoS ONE 9, e109085 (10.1371/journal.pone.0109085) 25275450 PMC4183542

[9] Erezyilmaz DF 2006 Imperfect eggs and oviform nymphs: a history of ideas about the origins of insect metamorphosis. Integr. Comp. Biol. 46, 795–807. (10.1093/icb/icl033) 21672785

[10] Reynolds S 2019 Cooking up the perfect insect: Aristotle's transformational idea about the complete metamorphosis of insects. Phil. Trans. R. Soc. B 374, 20190074 (10.1098/rstb.2019.0074) 31438815 PMC6711290

[11] Heming BS 2003 Insect development and evolution. Ithaca, NY: Cornell University Press.

[12] Jindra M 2019 Where did the pupa come from? The timing of juvenile hormone signalling supports homology between stages of hemimetabolous and holometabolous insects. Phil. Trans. R. Soc. B 374, 20190064 (10.1098/rstb.2019.0064) 31438814 PMC6711293

[13] Truman JW, Riddiford LM 2002 Endocrine insights into the evolution of metamorphosis in insects. Annu. Rev. Entomol. 47, 467–500. (10.1146/annurev.ento.47.091201.145230) 11729082

[14] Yang AS 2001 Modularity, evolvability, and adaptive radiations: a comparison of hemi- and holometabolous insects. Evol. Dev. 3, 59–72. (10.1046/j.1525-142x.2001.003002059.x) 11341675

[15] Shepherd D, Bate CM 1990 Spatial and temporal patterns of neurogenesis in the embryo of the locust (*Schistocerca gregaria*). Development 108, 83–96.

[16] Truman JW 1996 Insect nervous system metamorphosis. In Metamorphosis: postembryonic reprogramming of gene expression in amphibian and insect cells (eds Gilbert LI, Tata JR, Atkinson BG), pp. 283–320. San Diego, CA: Academic Press.

[17] Paulus HF 1986 Evolutionswege zum Larvalauge der Insekten-ein Modell für die Entstehung und die Ableitung der ozellären Lateralaugen der Myriapoda von Fazettenaugen. Zool. Jahrb. Syst. 113, 353–371. (10.1111/j.1439-0469.1989.tb00345.x)

[18] Liu Z, Friedrich M 2004 The *Tribolium* homologue of *glass* and the evolution of insect larval eyes. Dev. Biol. 269, 36–54. (10.1016/j.ydbio.2004.01.012) 15081356

[19] Inoue Y, Mito T, Miyawaki K, Matsushima K, Shinmyo Y, Heanue TA, Mardon G, Ohuchi H, Noji S 2002 Correlation of expression patterns of *homothorax*, *dachshund*, and *Distal-less* with the proximodistal segmentation of the cricket leg bud. Mech. Dev. 113, 141–148. (10.1016/S0925-4773(02)00017-5) 11960702

[20] Erezyilmaz DF, Riddiford LM, Truman JW 2004 Juvenile hormone acts at embryonic molts and induces the nymphal cuticle in the direct-developing cricket. Dev. Genes Evol. 214, 313–323. (10.1007/s00427-004-0408-2) 15170568

[21] Tanaka K, Truman JW 2005 Development of the adult leg epidermis in *Manduca sexta*: contribution of different larval cell populations. Dev. Genes Evol. 215, 78–89. (10.1007/s00427-004-0458-5) 15647943

[22] Tanaka K, Truman JW 2007 Molecular patterning mechanism underlying metamorphosis of the thoracic leg in *Manduca sexta*. Dev. Biol. 305, 539–550. (10.1016/j.ydbio.2007.02.042) 17418115

[23] Kim C-W 1959 The differentiation center inducing development from larval to adult leg in *Pieris brassicae* (Lepidoptera). J. Embryol. Exp. Morphol. 7, 572–582. 14409080

[24] Truman JW, Hiruma K, Allee JP, MacWhinnie SGB, Champlin DT, Riddiford LM 2006 Juvenile hormone is required to couple imaginal disc formation with nutrition in insects. Science 312, 1385–1388. (10.1126/science.1123652) 16741122

[25] Thomas JB, Bastiani MJ, Bate M, Goodman CS 1984 From grasshopper to *Drosophila*: a common plan for neuronal development. Nature 310, 203–207. (10.1038/310203a0) 6462206

[26] Truman JW, Ball EE 1998 Patterns of embryonic neurogenesis in a primitive wingless insect, the silverfish, *Ctenolepisma longicaudata*: comparison with those seen in flying insects. Dev. Genes Evol. 208, 357–368. (10.1007/s004270050192) 9732550

[27] Cohen SM 1993 Imaginal disc development. In The development of Drosophila melanogaster, vol. 2 (eds Bate M, Martinez-Arias A.), pp. 747–841. Plainview, NY: Cold Spring Harbor Press.

[28] Konopova B, Zrzavý J 2005 Ultrastructure, development, and homology of insect embryonic cuticles. J. Morph. 267, 339–362. (10.1002/jmor.10338) 15838850

[29] Kato Y, Riddiford LM 1987 The role of 20-hydroxyecdysone in stimulating epidermal mitoses during the larval-pupal transformation of the tobacco hornworm, *Manduca sexta**.* Development 100, 227–236.

[30] Svácha P 1992 What are and what are not imaginal discs: reevaluation of some basic concepts (Insecta, Holometabola). Dev. Biol. 154, 101–117. (10.1016/0012-1606(92)90052-I) 1426619

[31] Huet C, Lenoir-Rousseux JJ 1976 Etude de la mise en place de la patte imaginale de *Tenebrio molitor.* I. Analyse expérimentale des processus de restauration au cours de la morphogenése. J. Embryol. Exp. Morph. 35, 303–321. (10.1016/0016-6480(75)90044-1) 939941

[32] Mirth CK, Truman JW, Riddiford LM 2009 The Ecdysone receptor controls the post-critical weight switch to nutrition-independent differentiation in *Drosophila* wing imaginal discs. Development 136, 2345–2353. (10.1242/dev.032672) 19515698 PMC2729347

[33] Champlin DT, Truman JW 1998 Ecdysteroids govern two phases of eye development during metamorphosis of the moth, *Manduca sexta*. Development 125, 2009–2018. 9570766 10.1242/dev.125.11.2009

[34] Erezyilmaz DF, Riddiford LM, Truman JW 2006 The pupal specifier *broad* directs progressive morphogenesis in a direct-developing insect. Proc. Natl Acad. Sci. USA 103, 6925–6930. (10.1073/pnas.0509983103) 16641104 PMC1458995

[35] Quennedey A, Quennedey B 1990 Morphogenesis of the wing Anlagen in the mealworm beetle *Tenebrio molitor* during the last larval instar. Tissue Cell 22, 721–740. (10.1016/0040-8166(90)90067-J) 18620327

[36] Clark-Hachtel CM, Linz DM, Tomoyasu Y 2013 Insights into insect wing origin provided by functional analysis of *vestigial* in the red flour beetle, *Tribolium castaneum*. Proc. Natl Acad. Sci. USA 110, 16 951–16 956. (10.1073/pnas.1304332110) PMC3801059 24085843

[37] Cheong SPS, Huang J, Bendena WG, Tobe SS, Hui JL 2015 Evolution of ecdysis and metamorphosis in arthropods: the rise of regulation of juvenile hormone. Integr. Comp. Biol. 55, 878–890. (10.1093/icb/icv066) 26105594

[38] Nijhout HF, Callier V 2014 Developmental mechanisms of body size and wing-body scaling in insects. Annu. Rev. Entomol. 60, 141–156. (10.1146/annurev-ento-010814-020841) 25341104

[39] Smykal V, Daimon T, Kayukawa T, Takaki K, Shinoda T, Jindra M 2015 Importance of juvenile hormone signaling arises with competence of insect larvae to metamorphose. Dev. Biol. 390, 221–230. (10.1016/j.ydbio.2014.03.006) 24662045

[40] Daimon T, Uchibori M, Nakao H, Sezutsu H, Shinoda T 2015 Knockout silkworms reveal a dispensable role for juvenile hormones in holometabolous life cycle. Proc. Natl Acad. Sci. USA 112, E4226–E4235. (10.1073/pnas.1506645112) 26195792 PMC4534237

[41] Aboulafia-Baginsky N, Pener M, Staal GB 1984 Chemical allatectomy of late *Locusta* embryos by a synthetic precocene and its effect on hopper morphogenesis. J. Insect Physiol. 30, 839–852. (10.1016/0022-1910(84)90057-X)

[42] Zhou X, Truman JW, Riddiford LM 2004 Overexpression of *broad*: a new insight into its role in the *Drosophila* prothoracic gland cells. J. Exp. Biol. 207, 1151–1161. (10.1242/jeb.00855) 14978057

[43] MacWhinnie SGB, Allee JP, Nelson CA, Riddiford LM, Truman JW, Champlin DT 2005 The role of nutrition in creation of the eye imaginal disc and initiation of metamorphosis in *Manduca sexta*. Dev. Biol. 285, 285–297. (10.1016/j.ydbio.2005.06.021) 16099447

[44] Koyama T, Syropyatova MO, Riddiford LM 2008 Insulin/IGF signaling regulates the change in commitment in imaginal discs and primordia by overriding the effect of juvenile hormone. Dev. Biol. 324, 258–265. (10.1016/j.ydbio.2008.09.017) 18845136

[45] Riddiford LM 1978 Ecdysone-induced change in cellular commitment of the epidermis of the tobacco hornworm, *Manduca sexta*, at the initiation of metamorphosis. Gen. Comp. Endocrin. 34, 438–446. (10.1016/0016-6480(78)90284-8) 648872

[46] Williams CM 1961 The juvenile hormone. II. Its role in the endocrine control of molting, pupation, and adult development in the Cecropia silkworm. Biol. Bull. 121, 572–585. (10.2307/1539456)

[47] Zhou X, Riddiford LM 2002 Broad specifies pupal development and mediates the ‘status quo’ action of juvenile hormone on the pupal-adult transformation in *Drosophila* and *Manduca**.* Development 129, 2259–2269. 11959833 10.1242/dev.129.9.2259

[48] Bergot BJ, Baker FC, Cerf DC, Jamieson G, Schooley DA 1981 Qualitative and quantitative aspects of juvenile hormone titers in developing embryos of several insect species: discovery of a new JH-like substance extracted from eggs of *Manduca sexta*. In Juvenile hormone biochemistry. Action, agonism and antagonism (eds Pratt GE, Brookes GT), pp. 33–45. Amsterdam, The Netherlands: Elsevier/North-Holland Biomedical Press.

[49] Riddiford LM, Williams CM 1967 The effects of juvenile hormone analogues on the embryonic development of silkworms. Proc. Natl Acad. Sci. USA 57, 595–601. (10.1073/pnas.57.3.595) 16591505 PMC335550

[50] Lagueux M, Hetru C, Goltzene F, Kappler C, Hoffmann JA 1979 Ecdysone titre and metabolism in relation to cuticulogenesis in embryos of *Locusta migratoria*. J. Insect Physiol. 25, 709–723. (10.1016/0022-1910(79)90123-9)

[51] Temin G, Zander M, Roussel JP 1986 Physico-chemical (GC-MS) measurements of juvenile hormone III titres during embryogenesis of *Locusta migratoria*. Int. J. Invert. Reprod. 9, 105–112. (10.1080/01688170.1986.10510184)

[52] Imboden H, Lanzrein B, Delbecque JP, Lüscher M 1978 Ecdysteroids and juvenile hormone during embryogenesis in the ovoviviparous cockroach *Nauphoeta cinerea*. Gen. Comp. Endocrinol. 36, 628–635. (10.1016/0016-6480(78)90104-1) 571384

[53] Maestro O, Cruz J, Pascual N, Martin D, Belles X 2005 Differential expression of two RXR/ultraspiracle isoforms during the life cycle of the hemimetabolous insect *Blattella germanica* (Dictyoptera, Blattellidae). Mol. Cell Endocrinol. 238, 27–37. (10.1016/j.mce.2005.04.004) 15953509

[54] Maestro JL, Pascual N, Treiblmayr K, Lozano J, Belles X 2010 Juvenile hormone and allatostatins in the German cockroach embryo. Insect Biochem. Mol. Biol. 40, 660–665. (10.1016/j.ibmb.2010.06.006) 20542115

[55] Fernandez-Nicolas A, Belles X 2017 Juvenile hormone signaling in short germ-band hemimetabolan embryos. Development 144, 4637–4644. (10.1242/dev.152827) 29122840

[56] Pecasse F, Beck Y, Ruiz C, Richards G 2000 *Krüppel-homolog*, a stage-specific modulator of the prepupal ecdysone response, is essential for *Drosophila* metamorphosis. Dev. Biol. 221, 53–67. (10.1006/dbio.2000.9687) 10772791

[57] Minakuchi C, Namiki T, Shinoda T 2009 *Krüppel homolog 1*, an early juvenile hormone-response gene downstream of *Methoprene-tolerant*, mediates its anti-metamorphic action in the red flour beetle *Tribolium castaneum**.* Dev. Biol. 325, 341–350. (10.1016/j.ydbio.2008.10.016) 19013451

[58] Lozano J, Belles X 2011 Conserved repressive function of Krüppel homolog 1 on insect metamorphosis in hemimetabolous and holometabolous species. Sci. Rep. 1, 163 (10.1038/srep00163) 22355678 PMC3240953

[59] Kayukawa T et al. 2014 Hormonal regulation and developmental role of Krüppel homolog 1, a repressor of metamorphosis, in the silkworm *Bombyx mori*. Dev. Biol. 388, 48–56. (10.1016/j.ydbio.2014.01.022) 24508345

[60] Minakuchi C, Namiki T, Yoshiyama M, Shinoda T 2008 RNAi-mediated knockdown of *juvenile hormone acid O-methyltransferase* gene causes precocious metamorphosis in the red flour beetle *Tribolium castaneum**.* FEBS J. 275, 2919–2931. (10.1111/j.1742-4658.2008.06428.x) 18435763

[61] Ureña E, Chafino S, Manjón C, Franch-Marro X, Martin D 2016 The occurrence of the holometabolous pupal stage requires the interaction between E93, Krüppel-homolog 1 and Broad-Complex. PLoS Genet. 12, e1006020 (10.1371/journal.pgen.1006020) 27135810 PMC4852927

[62] Bayer C, von Kalm L, Fristrom JW 1996 Gene regulation in imaginal disc and salivary gland development during *Drosophila* metamorphosis. In Metamorphosis: postembryonic reprogramming of gene expression in amphibian and insect cells (eds Gilbert LI, Tata JR, Atkinson BG), pp. 321–361. San Diego, CA: Academic Press.

[63] Zhou B, Hiruma K, Shinoda T, Riddiford LM 1998 Juvenile hormone prevents ecdysteroid-induced expression of Broad Complex RNAs in the epidermis of the tobacco hornworm, *Manduca sexta**.* Dev. Biol. 203, 233–244. (10.1006/dbio.1998.9059) 9808776

[64] Ijiro T, Urakawa H, Yasukochi Y, Takeda M, Fujiwara Y 2004 cDNA cloning, gene structure, and expression of Broad-Complex (BR-C) genes in the silkworm, *Bombyx mori**.* Insect Biochem. Mol. Biol. 34, 963–969. (10.1016/j.ibmb.2004.06.005) 15350615

[65] Konopova B, Jindra M 2008 Broad-Complex acts downstream of Met in juvenile hormone signaling to coordinate primitive holometabolan metamorphosis. Development 135, 559–568. (10.1242/dev.016097) 18171683

[66] Suzuki Y, Truman JW, Riddiford LM 2008 The role of Broad in the development of *Tribolium castaneum*: implications for the evolution of the holometabolous insect pupa. Development 135, 569–577. (10.1242/dev.015263) 18171684

[67] Piulachs M-D, Pagone V, Belles X 2010 Key roles of the *Broad-Complex* gene in insect embryogenesis. Insect Biochem. Mol. Biol. 40, 468–475. (10.1016/j.ibmb.2010.04.006) 20403438

[68] Kiss I, Beaton AH, Tardiff J, Fristrom D, Fristrom JW 1988 Interactions and developmental effect of mutations in the *Broad-Complex* of *Drosophila melanogaster**.* Genetics 118, 247–259. 3129334 10.1093/genetics/118.2.247 PMC1203278

[69] Ureña E, Manjón C, Franch-Marro X, Martin D 2014 Transcription factor E93 specifies adult metamorphosis in hemimetabolous and holometabolous insects. Proc. Natl Acad. Sci. USA 111, 7024–7029. (10.1073/pnas.1401478111) 24778249 PMC4024875

[70] Kayukawa T, Jouraku A, Ito Y, Shinoda T 2017 Molecular mechanism underlying juvenile hormone-mediated repression of precocious larval-pupal metamorphosis. Proc. Natl Acad. Sci. USA 114, 1057–1062. (10.1073/pnas.1615423114) 28096379 PMC5293048

[71] Chafino S, Ureña E, Casanova J, Casacuberta E, Franch-Marro X, Martin D 2019 Upregulation of *E93* gene expression acts as the trigger for metamorphosis independently of the threshold size in the beetle *Tribolium castaneum**.* Cell Rep. 27, 1039–1049. (10.1016/j.celrep.2019.03.094) 31018122

[72] Duncan DM, Kiefel P, Ducan I 2017 Mutants for *Drosophila* 3b are defective in mitochondrial function and larval cell death. G3-Genes, Genomes Genet. 7, 789–799. (10.1534/g3.116.037366) PMC5345709 28104670

[73] Uyehara CM, Nystrom SL, Niederhuber MJ, Leatham-Jensen M, Ma Y, Buttitta LA, McKay DJ 2017 Hormone-dependent control of developmental timing through regulation of chromatin accessibility. Genes Dev. 31, 862–875. (10.1101/gad.298182.117) 28536147 PMC5458754

[74] Konopova B, Smykal V, Jindra M 2011 Common and distinct roles of juvenile hormone signaling genes in metamorphosis of holometabolous and hemimetabolous insects. PLoS ONE 6, e28728 (10.1371/journal.pone.0028728) 22174880 PMC3234286

[75] Huang J-H, Lozano J, Belles X 2013 Broad-complex functions in postembryonic development of the cockroach *Blattella germanica* shed new light on the evolution of insect metamorphosis. Biochim. Biophys. Acta 1830, 2178–2187. (10.1016/j.bbagen.2012.09.025) 23041750

[76] Erezyilmaz DF, Rynerson MR, Truman JW, Riddiford LM 2009 The role of the pupal determinant *broad* during embryonic development of a direct-developing insect. Dev. Genes Evol. 219, 535–544. (10.1007/s00427-009-0315-7) 20127251 PMC2884998

[77] Belles X, Santos CG 2014 The MEKRE93 (Methoprene tolerant-Kruppel homolog 1-E93) pathway in the regulation of insect metamorphosis, and the homology of the pupal stage. Insect Biochem. Mol. Biol. 52, 60–68. (10.1016/j.ibmb.2014.06.009) 25008785

[78] Buaklin A, Sittikankaew K, Khamnamtong B, Menasveta P, Klinbunga S 2013 Characterization and expression analysis of the *Broad-complex (Br-c)* gene of the giant tiger shrimp *Penaeus monodon**.* Comp. Biochem. Physiol. B 164, 280–289. (10.1016/j.cbpb.2013.02.004) 23485783

[79] Jiang SF, Zhang YP, Sun SM, Gong YS, Xiong YW, Qiao H, Zhang WY, Jin SB, Fu HT 2015 Molecular cloning, characterization, and expression analysis of a Broad-Complex homolog during development in the oriental river prawn *Macrobrachium nipponense*. Genet. Mol. Res. 14, 5141–5152. (10.4238/2015.May.18.4) 26125707

[80] Zhou B, Williams DW, Altman J, Riddiford LM, Truman JW 2009 Temporal patterns of *broad* isoform expression during the development of neuronal lineages in *Drosophila**.* Neural Dev. 4, 39 (10.1186/1749-8104-4-39) 19883497 PMC2780399

[81] Starck JM, Ricklefs RE 1998 Patterns of development: the altricial–precocial spectrum. In Avian growth and development: evolution within the altricial-precocial spectrum (eds Starck JM, Ricklefs RE), pp. 3–30. Oxford, UK: Oxford University Press.

[82] Minakuchi C, Tanaka M, Miura K, Tanaka T 2011 Developmental profile and hormonal regulation of the transcription factors *broad* and *Krüppel homolog 1* in hemimetabolous thrips. Insect Biochem. Mol. Biol. 41, 125–134. (10.1016/j.ibmb.2010.11.004) 21111817

[83] Vea IM, Tanaka S, Shiotsuki T, Jouraku A, Tanaka T, Minakuchi C 2016 Differential juvenile hormone variations in scale insect extreme sexual dimorphism. PLoS ONE 11, e0149459 (10.1371/journal.pone.0149459) 26894583 PMC4760703

[84] Bernays EA 1971 The vermiform larva of *Schistocerca gregaria* (Forskal): form and activity (Insects, Orthoptera). Z. Morph. Tiere 70, 183–200.

[85] Ishimaru Y, Tomonari S, Watanabe T, Noji S, Mito T 2019 Regulatory mechanisms underlying the specification of the pupal-homologous stage in a hemimetabolous insect. Phil. Trans. R. Soc. B 374, 20190225 (10.1098/rstb.2019.0225) 31438810 PMC6711289

[86] Vea IM, Tanaka S, Tsuji T, Shiotsuki T, Jouraku A, Minakuchi C 2019 *E93* expression and links to the juvenile hormone in hemipteran mealybugs with insights on female neoteny. Insect Biochem. Mol. Biol. 104, 65–72. (10.1016/j.ibmb.2018.11.00) 30503224

